# Association of Glycosylated Hemoglobin Level and Cancer-Related Mortality in Patients without Diabetes

**DOI:** 10.3390/jcm11195933

**Published:** 2022-10-08

**Authors:** Tae Kyung Yoo, Mi Yeon Lee, Sul A. Lee, Eun Sun Cheong, Mi Hae Seo, Ki Chul Sung

**Affiliations:** 1 Department of Medicine, MetroWest Medical Center, Framingham, MA 01702, USA; 2 Division of Biostatistics, Department of R&D Management, Kangbuk Samsung Hospital, Sungkyunkwan University School of Medicine, Seoul 03181, Korea; 3 Nephrology Division, Brigham and Women’s Hospital, Boston, MA 02115, USA; 4 Division of Cardiology, Department of Internal Medicine, Seoul Eulji Hospital, Eulji University School of Medicine, Seoul 01830, Korea; 5 Department of Internal Medicine, Soonchunhyang University Gumi Hospital, Gumi 39371, Korea; 6 Division of Cardiology, Department of Internal Medicine, Kangbuk Samsung Hospital, Sungkyunkwan University School of Medicine, Seoul 03181, Korea

**Keywords:** HbA1c, cancer, mortality, prediabetes

## Abstract

Background: Previous studies have reported that abnormal glucose metabolism is associated with poor cancer outcomes. Glycated hemoglobin A1c (HbA1c) is an important indicator of glucose metabolism. This study aimed to investigate the relationship between nondiabetic HbA1c levels and cancer-related mortality. Methods: This was a retrospective cohort study of Koreans who attended an annual or biennial health checkup program. The study group was categorized based on the quintile of HbA1c level (Q1, 3.0–5.1%; Q2, 5.2–5.3%; Q3, 5.4%; Q4, 5.5–5.6%, Q5, 5.7–6.4%). Cancer-related mortality was determined using the mortality data from the Korea National Statistical Office. Participants with an established diagnosis of diabetes or cancer were excluded. Cancer-related mortality was assessed depending on each HbA1c level with adjustment for factors that could influence mortality. Results: A total of 589,457 participants were included in this study. During a median follow-up duration of 6.99 years, 1712 cancer-related deaths were reported. The risk of cancer-related mortality was significantly higher in the Q5 group (hazard ratio (HR) 1.23, range 1.02–1.47 in model 1; HR 1.25, range 1.04–1.50 in model 2). HbA1c levels were linearly associated with cancer-related deaths (Ptrend = 0.021 in model 1; 0.013 in model 2). HbA1c level and colorectal, stomach, and lung cancer mortality exhibited a positive relationship, whereas liver cancer-related mortality showed an inverse relationship with HbA1c level (Ptrend = 0.001). Conclusions: Our study showed that abnormal glucose metabolism is significantly associated with cancer-related mortality, and its relationship varies with each type of cancer.

## 1. Introduction

Diabetes and cancer are very prevalent diseases worldwide, which seriously affect patients’ health status [1]. In 2020, an estimated 19.3 million new cancer cases and 10 million cancer deaths were reported worldwide [2]. Meanwhile, newly diagnosed cancer cases and deaths from cancer were predicted as 243,263 and 80,546, respectively, with lung, stomach, thyroid, colorectal, and breast cancers as the most common types of cancer in Korea [3]. Abnormal glucose metabolism is a widespread chronic condition worldwide [4]. In 2018, 13.8% of Korean adults aged ≥30 years had diabetes, and the prevalence of impaired fasting glucose was 26.9% in adults aged ≥30 years [5].

Glycated hemoglobin A1c (HbA1c) is an established indicator of average blood glucose concentrations for 2–3 months, with low within-person variability [6]. In addition, HbA1c assays are well-standardized [7]. An HbA1c level of 48 mmol/mol (6.5%) has been approved for the diagnosis of type 2 diabetes [8]. Large epidemiologic studies have demonstrated strong links between HbA1c level and complications, with stronger associations than those observed for standard glucose measures, leading HbA1c levels to become a treatment target for diabetes control [9].

Although diabetes and cancer share many common risk factors [10], existing studies on the relationship between HbA1c level and cancer have reported conflicting results [11,12]. Goto et al. reported that higher HbA1c levels in Japanese individuals without known diabetes are associated with the risk of all types of cancers, but liver cancer incidence showed an inverse relationship [12]. A recent study using the UK biobank reported that, apart from pancreatic cancer, no positive association between HbA1c levels and cancer risk was found [13]. Another study conducted in a Chinese population reported no relationship between HbA1c levels and cancer incidence [14].

Furthermore, previous studies on HbA1c levels and cancer mortality have also been conflicting. Multiple studies have reported that HbA1c level is associated with an increased risk of poor cancer outcomes at several anatomical sites, including the breast and colon [15,16,17,18]. A study conducted among Singaporean Chinese also reported that HbA1c was related to cancer mortality [19]. However, those studies included confirmed type 2 diabetes cases or HbA1c levels ≥ 6.5%. Although a prospective cohort study among Japanese workers showed that the prediabetes population was associated with a significantly increased risk of death from cancer [20], only a few studies have examined the relationship between HbA1c levels and cancer mortality in a population without diabetes [21]. Therefore, this study aimed to investigate the relationship between HbA1c levels and cancer-related mortality among a population without a previous history of cancer and diabetes.

## 2. Materials and Methods

### 2.1. Study Population and Study Period

This retrospective study was performed using a subsample from the Kangbuk Samsung Health Study. This was a cohort study of Korean men and women who attended an annual or biennial health checkup program at one of the Kangbuk Samsung Hospital Total Healthcare Centers in Seoul and Suwon, South Korea, between 2 March 2005, and 29 December 2018 (*n* = 627,315). The institutional review board of Kangbuk Samsung Hospital approved this study (IRB no. KBSMC 2013-01-010-018). In addition, the requirement for informed consent was waived due to the following reasons: the use of anonymized retrospective data collected as a routine part of the health checkup program, and all data were already linked to mortality data from the Korea National Statistical Office (KNSO).

KNSO is a national civil registry for death, containing information about the age, causes, date, and time of death. KNSO provides valid information as it is mandatory to report the death to the local government according to the family register law and the Statistics law regardless of the place of death in South Korea [22]. In South Korea, physicians are legally required to complete death certificates, which include information on the disease directly leading to death, antecedent causes, and other major conditions contributing to death [22]. Each local government sends the data to the KNOS, where all the transmitted data are reviewed [22,23]. The cause of death is classified and coded according to the World Health Organization definition and ICD-10 [23]. The coding is reviewed and finalized by the committee. Nationally, almost 100% of deaths were certified by 2007 [22].

### 2.2. Laboratory Assays

Blood samples were collected from the antecubital vein after at least a 10-h fast for HbA1c and fasting glucose (mg/dL) levels during the health checkup. HbA1c levels were measured using a turbidimetric inhibition immunoassay with a Cobas c513 analyzer (Roche Diagnostics, Tokyo, Japan), with a reference value <5.7%, following the International Federation of Clinical Chemistry methods, the Diabetes Control and Complications Trial standards, and the National Glycohemoglobin Standardization Program. The intra-assay coefficient of variation was 2.3%, and the inter-assay coefficient of variation was 2.4% during the study period. Serum glucose level was measured using a Cobas Integra 800 apparatus (Roche Diagnostics, Basel, Switzerland), using the hexokinase method. HbA1c collected at study inclusion was used for the analysis. The Kangbuk Samsung Hospital’s Laboratory Medicine Department in Seoul, Korea, has been accredited by the Korean Association of Quality Assurance for Clinical Laboratories and the Korean Society of Laboratory Medicine. In addition, the laboratory participated in the College of American Pathologists’ Survey and Proficiency Testing program.

### 2.3. Cancer Outcomes

Cancer-related mortality was defined as the direct cause of death based on the International Classification of Disease (ICD)-Tenth Edition code for cancer from the KNSO. All cancer-related death was assessed. In addition, the cancer-related deaths owing to common cancer types in Korea were considered primary outcomes of interest, including stomach (C16), lung (C34), colorectal (C18–21), breast cancer (C50), and liver cancer (C22), as well as cervical cancer (C53) and prostate cancer (C61), which were also prevalent in women and men. Pancreatic cancer-related death according to sub-diabetic HbA1c level was assessed in the previous study using the Kangbuk Samsung Health Study [24]. We conducted an analysis for pancreatic cancer (C25)-related mortality in our cohort to compare with other cancer mortalities.

### 2.4. Covariates

The following variables were extracted from the health checkup results and self-reports during the checkup: demographic and socioeconomic data (age, sex, education level (university, community college graduate or higher)), BMI (kg/m^2^, continuous), lifestyle behaviors such as smoking status (current smoker, former smoker, nonsmoker) and alcohol consumption (g/day), regular exercise (75 min per week engaging in vigorous physical activities and 150 min per week of moderate physical activities), and lab results (fasting glucose (mg/dL) and HbA1c (%) levels) [25]. Current smokers were defined as those who smoked more than 5 packs (more than 100 cigarettes) in their lifetime and who currently smoked cigarettes [26]. Former smokers were defined as those who had smoked more than 100 cigarettes in their lifetime but who had quit smoking at the time of checkup [26]. The health questionnaire conforms to international standards and uses standardized questionnaire tools in Korea.

The questionnaire incorporates multi-level questions for each condition to ensure that participants provide detailed medical history. For example, diabetes-related questionnaires are constructed as follows. If a participant answered “Yes” to the question “Have you ever been medically diagnosed with diabetes by your doctor?” a participant was directed to answer the next question, “if you were ever diagnosed with diabetes, what is the current status?”. Participants could choose one of the four answers, including currently under treatment, underwent treatment in the past but not currently, never underwent treatment, or treatment terminated. Those who answered “Yes” to an initial question about diabetes were considered to have a history of diabetes, regardless of their current treatment status. Those who responded “Yes” to an initial inquiry and checked “currently under treatment” were counted as a diabetes on medical treatment and were excluded from the analysis. For the history of cancer, there was a separate sheet in the questionnaire. The first question was “Have you ever been diagnosed with cancer?” and if a participant answered “Yes”, a participant was directed to mark the type of cancer diagnosed and then the diagnosis age. If a participant answered “Yes” to an initial question about cancer, they were considered to have a history of cancer and excluded from the analysis. Among 627,315 participants eligible for study inclusion, fasting glucose level (*n* = 6, 0.00%), alcohol consumption (*n* = 38,473, 0.06%), and BMI (*n* = 158, 0.03%) were missing.

### 2.5. Statistical Analysis

Continuous variables were expressed as mean ± standard deviation. When variables had right-skewed distribution, the variable was expressed as median (interquartile range). Categorical variables are expressed as frequency (%). One-way ANOVA was used to compare continuous variables. Pearson’s chi-square test was used to compare categorical variables, which were expressed as percentages. A Cochran–Armitage test was performed to evaluate the linear trend in the HbA1c group. Cox’s proportional-hazards model was used to assess the hazard of cancer-related mortality depending on each HbA1c group (Q1–Q5, Q1 as a reference group) after adjusting for sex, age, BMI, regular exercise, alcohol consumption, current smoking status, and level of education (model 1). In model 2, further adjustments were made for fasting blood glucose levels ≥ 126 mg/dL. Category numbers were used as a continuous variable to test for the linear trend of hazard ratio. In addition, we conducted a subgroup analysis using multiplicative interaction in the Cox regression model. The Cox modeling excluded each corresponding subgroup variable during the subgroup analysis. In addition, we carried out a separate analysis using participants age < 40 group and ≥40 group to assess the impact of age on the association. Hazard ratios (HRs) and 95% CI were estimated. The log-rank test was used to draw the Kaplan–Meier survival curve. Two-sided *p* values were calculated, and the threshold for significance was set at *p* < 0.05. STATA (version 17.0, StataCorp LP, College Station, TX, USA) was used for statistical analysis.

## 3. Results

### 3.1. Cohort Description

**Figure 1:**
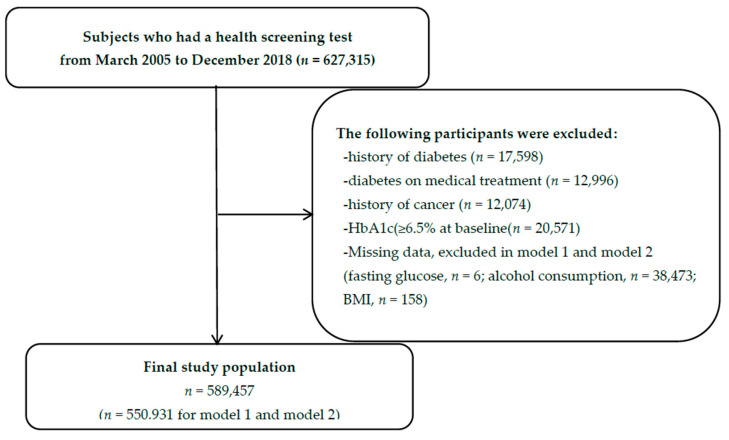
Flow chart for the selection of study subjects.

Of the 627,315 participants in a cohort study that measured HbA1c, a total of 37,858 participants were excluded because of a history of diabetes (*n* = 17,598), diabetes on medical treatment (*n* = 12,996), history of cancer (*n* = 12,074), and diabetic levels of HbA1c (≥6.5%) at baseline (*n* = 20,571). After exclusion, 589,457 participants were included for the analysis. Due to the missing variables (*n* = 38,526), 550,931 participants were included in the adjusted analysis (model 1 and model 2) (Figure 1). Those excluded from the analysis had higher age, male proportion, BMI, fasting glucose level, and smoking rate than included participants (Supplemental Table S1).

Table 1 presents the baseline characteristics of the participants. Participants were categorized to five groups based on HbA1c quintiles. HbA1c was measured to 1 decimal, and the following categorization was carried out due to the HbA1c values on cut-off: HbA1c levels 3.0–5.1% (*n* = 79,234, 13.44%), 5.2–5.3% (*n* = 127,430, 21.62%), 5.4% (*n* = 82,849, 14.06%), 5.5–5.6% (*n* = 153,392, 26.02%), and 5.7–6.4% (*n* = 146,552, 24.86%). The number of participants in HbA1c 5.1–5.7% range is provided in a Supplemental Table S2.

Individuals with higher HbA1c levels were observed to have an increased age, higher male percentage, higher BMI, higher fasting glucose level, higher physical activity level, and lower college education rates (Ptrend < 0.001). In addition, participants with higher HbA1c quintile showed a higher rate of progression to diabetes (Supplemental Table S3).

### 3.2. Cancer Mortality

Among 589,457 individuals with a median follow-up of 6.99 years for a total of 4,213,135.7 person-years (PY), 1,712 (0.29%) individuals had cancer-related mortalities (incidence rate, 4.06 per 10^4^ PY); lung cancer (ICD topology code: C34), 372 cases (incidence rate, 0.88 per 10^4^ PY); liver cancer (C22), 215 cases (incidence rate, 0.51 per 10^4^ PY); stomach cancer (C16), 172 cases (incidence rate, 0.41 per 10^4^ PY); colorectal cancer (C18–21), 118 cases (incidence rate, 0.28 per 10^4^ PY); breast cancer (C50), 70 cases (incidence rate, 0.17 per 10^4^ PY); prostate cancer (C61), 25 cases (incidence rate, 0.06 per 10^4^ PY); cervical cancer (C53), 16 cases (incidence rate, 0.04 per 10^4^ PY); and other sites, 720 cases (incidence rate, 1.71 per 10^4^ PY).

**Figure 2:**
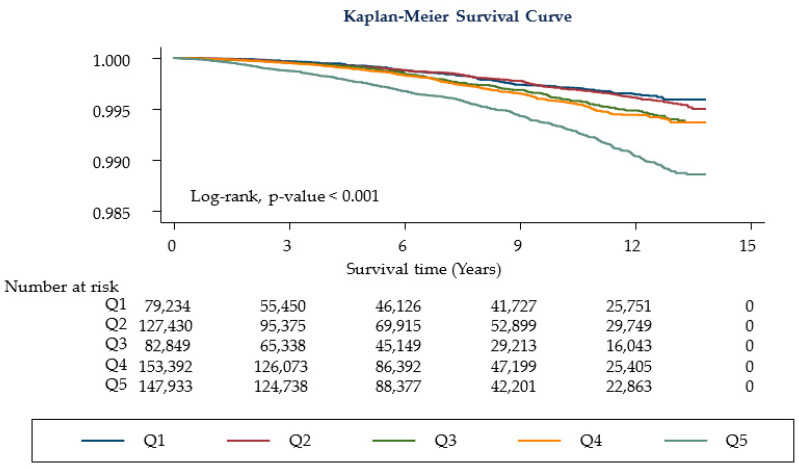
Kaplan-Meier curves for study participants. Q1: HbA1c levels 3.0–5.1% (*n* = 79,234, 13.44%), Q2: 5.2–5.3% (*n* = 127,430, 21.62%), Q3: 5.4% (*n* = 82,849, 14.06%), Q4: 5.5–5.6% (*n* = 153,392, 26.02%), Q5: 5.7–6.4% (*n* = 146,552, 24.86%).

Table 2 shows the cancer-related mortality according to the HbA1c category. Compared with participants with lower HbA1c levels (Q1–Q4, HbA1c level 3.0–5.6%), those with higher levels of HbA1c (Q5) had a higher risk of all-cancer-related mortality (HR 1.19; CI 1.00–1.41). This tendency remained constant after the adjustment (model 1, HR 1.23; CI 1.02–1.47, Ptrend = 0.021 and model 2, HR 1.25; CI 1.04–1.50; Ptrend = 0.013). Even after excluding death within <1 year of follow-up, the higher HbA1c level group (Q5) consistently showed increased cancer mortality (model 1, HR 1.21; CI 1.01–1.46; Ptrend = 0.045 and model 2, HR 1.23; CI 1.02–1.49; Ptrend = 0.027, Figure 2). When we assessed the relationship in the age < 40 group, there was no significant difference in mortality among the HbA1c quintile (Supplemental Table S4). However, when we assessed the relationship in age ≥ 40 group, those with higher levels of HbA1c (Q5) had a higher risk of all-cancer-related mortality (Supplemental Table S5), Table 3 shows the relationship between cancer-related mortality and HbA1c level. High HbA1c levels (Q5) were associated with a low risk of liver cancer-related mortality (HR 0.48; CI 0.30–0.76; Ptrend = 0.001 in model 1 and HR 0.49; CI 0.31–0.78; Ptrend = 0.001 in model 2), whereas higher HbA1c (Q5) levels were associated with an increased risk of lung cancer (model 1, HR 1.81; 95% CI 1.19–2.77; Ptrend = 0.002; model 2, HR 1.84; 95% CI 1.2–2.81; Ptrend = 0.002), colorectal cancer (model 1, HR 3.30; 95% CI 1.28–8.50; Ptrend = 0.017; model 2, HR 3.48; 95% CI 1.35–8.98; Ptrend = 0.011), pancreatic cancer (model 1, HR 2.06; 95% CI 1.07–3.99; model 2, HR 1.99; 95% CI 1.02–3.86). Stomach cancer showed an increasing trend of association in the higher HbA1c quintile (model 1, Ptrend = 0.044; model 2, Ptrend = 0.034).

In addition, prostate cancer risk was highest in the Q3 group, trending down from the Q3 to Q5 group, but was not statistically significant (model 1, Ptrend = 0.087; model 2, Ptrend = 0.064). Breast cancer- and cervical cancer-related mortality did not show any statistically significant correlations with HbA1c level. Table 4 shows the results of the subgroup analysis. In all models, the subgroup with less physical activity showed significantly increased cancer-related mortality in the Q5 group (model 1, Pinter = 0.038; model 2, Pinter = 0.038). When we carried out the same analysis in the age < 40 group, there was no significant difference in mortality between each HbA1c quintile and cancer mortality (Supplemental Table S6). However, when we assessed the relationship in the age ≥ 40 group, Q5 group showed an increased risk of lung cancer, colorectal cancer, pancreatic cancer mortality, and a lower risk of liver cancer mortality (Supplemental Table S7).

## 4. Discussion

This study showed that higher HbA1c levels within the nondiabetic range (5.7–6.4%) were independently associated with increased cancer-related mortality, especially in colorectal and lung cancers. In contrast, an inverse relationship was observed between HbA1c levels and liver cancer-related mortality. Thus, the current study results suggest that glucose metabolism might have a differential association with each type of cancer.

In this study, after adjusting for common cancer risks, the HR for all cancer-related mortality in participants with high HbA1c levels (Q5, HbA1c level 5.7–6.4%) was significantly higher (model 1, HR 1.23; model 2, HR 1.25) than those with low HbA1c levels. These results support the notion that abnormal glucose metabolism can affect cancer mortality [27]. Moreover, this finding remained consistent after excluding death within 1 year. This result suggests that preexisting disease is not the cause of high mortality in the Q5 group. Of note, due to the age heterogeneity between the HbA1c quintile, we performed a separate analysis by dividing participants into age ≥ 40 and age < 40 groups. There was the same trend of association between the HbA1c quintile and cancer mortality in the ≥40 age group, while the <40 age group did not show a significant difference. No significant cancer mortality difference among HbA1c quintiles in the age < 40 group can be explained by a relatively small number of mortality.

Although previous studies evaluated the association between HbA1c levels and the development of lung [28] and colorectal cancers [29,30,31], they did not evaluate cancer-related mortality. In addition, they were limited by the relatively small sample sizes or case numbers or did not exclude the diabetic range of HbA1c.

Our finding is novel because we investigated the relationship between cancer-related mortality and nondiabetic levels of HbA1c. Moreover, our study had a larger sample size and excluded participants with preexisting diabetes and HbA1c levels ≥ 6.5%. Thus, our findings more reliably suggest that increased HbA1c levels, even in the nondiabetic range, can be associated with increased cancer-related mortality, especially in lung and colon cancers. In contrast, an inverse relationship was observed between liver cancer-related mortality and HbA1c levels.

Only a few studies have evaluated the association between Hba1c levels and the mortality of multiple cancers [20,32,33,34]. A previous study with 2,686 participants without a history of diabetes and a mean duration of follow-up of 7.54 ± 2.1 years failed to show any significant increase in cancer mortality in the HbA1c < 6.5% group [33]. However, the study lacked information about participants’ alcohol consumption and education level, raising concerns about residual confounding. In addition, most of the cohort in the study had coronary artery disease at baseline, raising concern about whether the study results can be applied to the general population.

In the Atherosclerosis Risk in Communities Study (ARIC) with 12,792 White and Black participants with a median follow-up of 15 years, cancer mortality was elevated among women at risk for diabetes (≥5.7–6.4% HR, 1.62; 96%CI, 1.24–2.11) [32]. In contrast, a significant difference in cancer mortality based on sex was not observed. Considering that HbA1c levels may vary with ethnicity [35], the different study populations in our study can explain these different results. Therefore, further studies incorporating more diverse ethnicities are needed to determine the association between HbA1c levels and cancer-related mortality.

The most significant group at risk in our study was the subgroup with HbA1c 5.7–6.4. This finding aligns with recent research that evaluated the relationship between prediabetes HbA1c level (5.7–6.4%) and cancer mortality in the Japanese population [20]. In addition, a study carried out in a pooled European cohort showed an increased risk of cancer death in the prediabetes population [36]. Several biologic mechanisms can explain this finding. Hyperglycemia in prediabetes can increase proinflammatory factor secretion, leading to oncogene expression and cancer metastasis [20,37,38]. Additionally, insulin resistance in prediabetes stimulates cancer cell proliferation, thereby increasing cancer mortality [36,39,40].

A unique result of this study was the relationship between HbA1c levels and liver cancer. Unlike other carcinomas, in liver cancer, the group with the lowest HbA1c level (Q1, 3–5.1%) had the highest cancer-related mortality, showing an inverse relationship. This can be caused by a decrease in HbA1c level because of increased destruction of RBCs caused by hypersplenism [41], which frequently accompanies liver cancer patients [12]. In addition, the liver is an organ where gluconeogenesis occurs [42,43]. As liver cancer progresses, hepatic gluconeogenesis is downregulated [44]. As a result, HbA1c levels, a marker of blood sugar level, might decrease with liver cancer progression.

Multiple studies have shown a varying relationship between gastric cancer-, prostate cancer-, breast cancer-, and cervical cancer-related mortalities and HbA1c levels or preexisting diabetes [45,46,47,48]. Our study showed a positive relationship between nondiabetic levels of HbA1c and gastric cancer-related mortality, whereas no association was found in those with prostate cancer, breast cancer, and cervical cancer. In addition, our study showed a significant association between the highest HbA1c quintile and pancreatic cancer mortality, consistent with a previous finding from Kangbuk Samsung Health Study [24]. Overall, these results suggest that abnormal glucose metabolism may have different relationships with each type of cancer.

One important finding of our subgroup analysis was that reduced physical activity was an independent risk factor for cancer mortality. This finding supports the previous notion that decreased physical activity is associated with increased cancer-related mortality [49].

The strengths of this study includes the large sample size and the large number of cancer cases, standardized HbA1c values, exclusion of preexisting diabetes and diabetic HbA1c level range, and the use of a nationwide database for cancer-related mortality. Despite these strengths, this study has several limitations. First, most of the participants were young white-collar workers in the Samsung group. Advanced age is an established risk factor for all cancers [50], which can affect cancer and cancer-related mortality among the study population. Second, it was a single-center study, and most participants were Korean, with a single ethnicity. Third, a history of hepatitis and cirrhosis was not recorded in the present study, which might confound the effect of glucose metabolism. Fourth, we used a self-report questionnaire to collect baseline medical history. Self-report bias should be considered. Fifth, participants with fasting glucose levels > 100 mg/dL did not have a confirmatory oral glucose tolerance test. It is possible that the diagnosis of diabetes was missed in this population. Sixth, some patients in the cohort developed diabetes during the follow-up period. As our study aimed to investigate the association between HbA1c level at baseline and cancer mortality, those who developed diabetes were not removed from the analysis. However, this could have affected our results. We tried to modify these limitations by adjusting fasting glucose level ≥ 126 mg/dL in model 2. Sixth, the association was seen only in the ≥40-year-old group, suggesting age as an effect modifier of the relationship. This needs to be considered in the interpretation of the result. Lastly, though we adjusted multiple potential confounders, other potential confounders may affect the results.

Despite these limitations, this study is novel. Unlike most previous studies, the association between nondiabetic HbA1c levels in participants without diabetes and cancer-related mortality was examined. Further prospective studies are needed to determine the effects of abnormal glucose metabolism and its association with cancer mortality.

## 5. Conclusions

In conclusion, higher HbA1c levels within the nondiabetic ranges in individuals without diabetes are associated with an increased risk of cancer-related mortality, and their relationship varies with each type of cancer.

## Figures and Tables

**Table 1:** Baseline Characteristics.

	Missing Data	Overall	HbA1c Category					*p* Value for Trend
			Q1 (3–5.1)	Q2 (5.2–5.3)	Q3 (5.4–5.4)	Q4 (5.5–5.6)	Q5 (5.7–6.4)	
Characteristics ^1^			*n* = 79,234	*n* = 127,430	*n* = 82,849	*n* = 153,392	*n* = 146,552	
Age (years)		39.25 ± 10.31	36 ± 7.86	36.79 ± 8.52	37.72 ± 9.23	39.27 ± 10.1	44.01 ± 11.88	<0.001
Male, *n* (%)		305,526 (51.83)	41,582 (52.48)	66,528 (52.21)	43,642 (52.68)	80,440 (52.44)	73,334 (50.04)	<0.001
BMI (kg/m^2^)	158	23.25 ± 3.3	22.53 ± 2.94	22.77 ± 3.06	22.99 ± 3.17	23.28 ± 3.28	24.18 ± 3.55	<0.001
Fasting glucose (mg/dL)	6	93.05 ± 9.05	89.68 ± 7.69	90.84 ± 7.69	91.85 ± 7.9	93.04 ± 8.15	97.46 ± 10.52	<0.001
Red blood cell count (10^6^/mm^3^)	14	4.75 ± 0.46	4.75 ± 0.48	4.75 ± 0.46	4.75 ± 0.46	4.75 ± 0.46	4.73 ± 0.45	<0.001
Alcohol amount grams (g/day)	38,473	5 (1–14)	6 (1–15)	5 (1–14)	5 (1–14)	5 (1–14)	4 (0–14)	0.896
Regular exercise		85,659 (14.53)	10,819 (13.65)	18,302 (14.36)	11,989 (14.47)	22,380 (14.59)	22,169 (15.13)	<0.001
Current smoker (%)		122,128 (20.72)	17,007 (21.46)	26,560 (20.84)	17,345 (20.94)	31,790 (20.72)	29,426 (20.08)	0.066
≥College graduate (%)		344,242 (58.4)	48,219 (60.86)	77,357 (60.71)	50,158 (60.54)	91,186 (59.45)	77,322 (52.76)	<0.001

Data are expressed as mean ± standard deviation or median (interquartile range). Abbreviations: BMI, body mass index. ^1^ Baseline characteristics were compared among groups using ANOVA test for continuous variables and Pearson’s Chi-square test for categorical variables.

**Table 2:** All cancer-related mortality according to HbA1c level of participants.

HbA1c Category	Person-Years	Cancer Mortality	Incidence Rate (per 10^4^ PY)	Age- and Sex Adjusted HR (95% CI)	Model 1	Model 2
Total period	4,213,135.7	1,712	4.06 (3.88–4.26)			
Q1	616,081.62	170	2.76 (2.37–3.21)	1 (reference)	1 (reference)	1 (reference)
Q2	917,985.11	262	2.85 (2.53–3.22)	1.03 (0.85–1.25)	1.02 (0.84–1.25)	1.02 (0.84–1.25)
Q3	581,155.08	210	3.61 (3.16–4.14)	1.2 (0.98–1.46)	1.22 (0.99–1.5)	1.22 (0.99–1.5)
Q4	1,067,645.1	401	3.76 (3.41–4.14)	1.07 (0.89–1.28)	1.04 (0.86–1.25)	1.04 (0.86–1.26)
Q5	1,030,268.8	669	6.49 (6.02–7)	1.19 (1.00–1.41)	1.23 (1.02–1.47)	1.25 (1.04–1.50)
*p* value for trend				0.043	0.021	0.013
Excluding death within 1 year cases	3,644,481.4	1634	4.48 (4.27–4.71)			
Q1	541,105.56	164	3.03 (2.6–3.53)	1 (reference)	1 (reference)	1 (reference)
Q2	796,118.44	255	3.2 (2.83–3.62)	1.05 (0.86–1.27)	1.03 (0.85–1.27)	1.04 (0.85–1.27)
Q3	501,217.89	202	4.03 (3.51–4.63)	1.21 (0.98–1.48)	1.22 (0.99–1.51)	1.23 (0.99–1.52)
Q4	918,578.41	384	4.18 (3.78–4.62)	1.08 (0.9–1.3)	1.04 (0.86–1.26)	1.05 (0.86–1.27)
Q5	887,461.14	629	7.09 (6.55–7.66)	1.19 (0.99–1.42)	1.21 (1.01–1.46)	1.23 (1.02–1.49)
*p* value for trend				0.060	0.045	0.027

Data are expressed as hazard ratios (95% confidence intervals). Model 1 was adjusted for sex, age, BMI (kg/m^2^, continuous), education level (university, community college graduate or higher), smoking status (smoker or nonsmoker), alcohol consumption (g/day), regular exercise. Model 2 was further adjusted for fasting glucose ≥ 126 mg/dL.

**Table 3:** Specific cancer-related mortality according to HbA1c level of participants.

HbA1c Category	Person-Years	Cancer Mortality	Incidence Rate (per 10^4^ PY)	Age- and Sex Adjusted HR (95% CI)	Model 1	Model 2
Lung cancer	4,213,135.7	372	0.88 (0.8–0.98)			
Q1	616,081.62	27	0.44 (0.3–0.64)	1 (reference)	1 (reference)	1 (reference)
Q2	917,985.11	47	0.51 (0.38–0.68)	1.19 (0.74–1.91)	1.17 (0.72–1.88)	1.17 (0.72–1.88)
Q3	581,155.08	43	0.74 (0.55–0.998)	1.52 (0.94–2.46)	1.48 (0.91–2.41)	1.48 (0.91–2.41)
Q4	1,067,645.1	72	0.67 (0.54–0.85)	1.16 (0.74–1.81)	1.11 (0.70–1.74)	1.11 (0.70–1.75)
Q5	1,030,268.8	183	1.78 (1.54–2.05)	1.88 (1.24–2.84)	1.81 (1.19–2.77)	1.84 (1.2–2.81)
*p* value for trend				0.001	0.002	0.002
Colorectal cancer	4,213,135.7	118	0.28 (0.23–0.34)			
Q1	616,081.62	5	0.08 (0.03–0.19)	1 (reference)	1 (reference)	1 (reference)
Q2	917,985.11	20	0.22 (0.14–0.34)	2.68 (1.01–7.16)	2.46 (0.91–6.62)	2.47 (0.91–6.65)
Q3	581,155.08	10	0.17 (0.09–0.32)	1.98 (0.67–5.79)	1.63 (0.53–4.99)	1.64 (0.54–5.03)
Q4	1,067,645.1	29	0.27 (0.19–0.39)	2.7 (1.04–7.01)	2.18 (0.82–5.80)	2.21 (0.83–5.88)
Q5	1,030,268.8	54	0.52 (0.4–0.68)	3.34 (1.31–8.51)	3.30 (1.28–8.50)	3.48 (1.35–8.98)
*p*-value for trend				0.018	0.017	0.011
Liver cancer	4,213,135.7	215	0.51 (0.45–0.58)			
Q1	616,081.62	33	0.54 (0.38–0.75)	1 (reference)	1 (reference)	1 (reference)
Q2	917,985.11	45	0.49 (0.37–0.66)	0.91 (0.58–1.43)	0.85 (0.53–1.36)	0.85 (0.53–1.36)
Q3	581,155.08	30	0.52 (0.36–0.74)	0.85 (0.52–1.39)	0.78 (0.46–1.31)	0.78 (0.46–1.32)
Q4	1,067,645.1	48	0.45 (0.34–0.60)	0.62 (0.40–0.98)	0.64 (0.40–1.02)	0.64 (0.41–1.02)
Q5	1,030,268.8	59	0.57 (0.44–0.74)	0.50 (0.32–0.78)	0.48 (0.30–0.76)	0.49 (0.31–0.78)
*p*-value for trend				<0.001	0.001	0.001
Stomach cancer	4,213,135.7	172	0.41 (0.35–0.47)			
Q1	616,081.62	18	0.29 (0.18–0.46)	1 (reference)	1 (reference)	1 (reference)
Q2	917,985.11	17	0.19 (0.12–0.30)	0.63 (0.33–1.23)	0.65 (0.33–1.28)	0.65 (0.33–1.29)
Q3	581,155.08	27	0.46 (0.32–0.68)	1.49 (0.82–2.70)	1.62 (0.87–2.99)	1.62 (0.88–3)
Q4	1,067,645.1	52	0.49 (0.37–0.64)	1.39 (0.81–2.39)	1.43 (0.81–2.52)	1.44 (0.82–2.53)
Q5	1,030,268.8	58	0.56 (0.44–0.73)	1.13 (0.65–1.95)	1.34 (0.76–2.37)	1.38 (0.78–2.44)
*p*-value for trend				0.151	0.044	0.034
Prostate cancer †	4,213,135.7	25	0.06 (0.04–0.09)			
Q1	616,081.62	0	-	N/A	N/A	N/A
Q2	917,985.11	2	0.02 (0.01–0.09)	1 (reference)	1 (reference)	1 (reference)
Q3	581,155.08	4	0.07 (0.03–0.18)	2.56 (0.47–14.04)	5.33 (0.59–47.82)	5.38 (0.6–48.24)
Q4	1,067,645.1	8	0.07 (0.04–0.15)	2.15 (0.45–10.26)	4.42 (0.55–35.84)	4.51 (0.56–36.56)
Q5	1,030,268.8	11	0.11 (0.06–0.19)	1.81 (0.39–8.38)	3.85 (0.48–30.76)	4.21 (0.53–33.66)
*p*-value for trend				0.160	0.087	0.064
Breast cancer ‡	4,213,135.7	70	0.17 (0.13–0.21)			
Q1	616,081.62	7	0.11 (0.05–0.24)	1 (reference)	1 (reference)	1 (reference)
Q2	917,985.11	12	0.13 (0.07–0.23)	1.05 (0.41–2.67)	1.13 (0.42–3.06)	1.13 (0.42–3.06)
Q3	581,155.08	10	0.17 (0.09–0.32)	1.37 (0.52–3.62)	1.29 (0.45–3.73)	1.29 (0.45–3.73)
Q4	1,067,645.1	22	0.21 (0.14–0.31)	1.56 (0.66–3.67)	1.36 (0.53–3.5)	1.36 (0.53–3.5)
Q5	1,030,268.8	19	0.18 (0.12–0.29)	1.18 (0.48–2.89)	1.33 (0.51–3.49)	1.36 (0.52–3.57)
*p* value for trend				0.498	0.498	0.465
Cervical cancer ‡	4,213,135.7	16	0.04 (0.02–0.06)			
Q1	616,081.62	3	0.05 (0.02–0.15)	1 (reference)	1 (reference)	1 (reference)
Q2	917,985.11	3	0.03 (0.01–0.1)	0.59 (0.12–2.93)	0.91 (0.15–5.44)	0.91 (0.15–5.44)
Q3	581,155.08	1	0.02 (0–0.12)	0.30 (0.03–2.92)	0.47 (0.04–5.17)	0.47 (0.04–5.18)
Q4	1,067,645.1	2	0.02 (0–0.07)	0.31 (0.05–1.88)	0.47 (0.06–3.38)	0.47 (0.07–3.39)
Q5	1,030,268.8	7	0.07 (0.03–0.14)	0.99 (0.24–4.09)	0.94 (0.17–5.28)	0.96 (0.17–5.4)
*p* value for trend				0.929	0.842	0.865
Pancreatic cancer	4,213,135.7	166	0.39 (0.34–0.46)			
Q1	616,081.62	12	0.19 (0.11–0.34)	1 (reference)	1 (reference)	1 (reference)
Q2	917,985.11	23	0.25 (0.17–0.38)	1.33 (0.66–2.67)	1.38 (0.67–2.85)	1.37 (0.67–2.84)
Q3	581,155.08	21	0.36 (0.24–0.55)	1.79 (0.88–3.65)	1.86 (0.89–3.89)	1.84 (0.88–3.86)
Q4	1,067,645.1	37	0.35 (0.25–0.48)	1.51 (0.78–2.91)	1.49 (0.75–2.96)	1.47 (0.74–2.93)
Q5	1,030,268.8	73	0.71 (0.56–0.89)	2.01 (1.07–3.77)	2.06 (1.07–3.99)	1.99 (1.02–3.86)
P for trend				0.022	0.028	0.044

Data are expressed as hazard ratios (95% confidence intervals). Model 1 was adjusted for sex, age, BMI (kg/m^2^, continuous), education level (university, community college graduate or higher), smoking status (smoker or nonsmoker), alcohol consumption (g/day), regular exercise. Model 2 was further adjusted for fasting glucose ≥ 126 mg/dL. † Male only. ‡ Female only.

**Table 4:** Cancer-related mortality in subgroup.

	HbA1c Category					*p*-Value for Interaction
	Q1	Q2	Q3	Q4	Q5	
Model 1						
Sex						0.316
Male	1 (reference)	1.09 (0.86–1.38)	1.24 (0.96–1.58)	1 (0.8–1.26)	1.23 (0.99–1.53)	
Female	1 (reference)	0.84 (0.58–1.23)	1.11 (0.75–1.63)	1.04 (0.73–1.46)	1.18 (0.84–1.65)	
Regular exercise						0.038
No	1 (reference)	1.07 (0.85–1.35)	1.36 (1.07–1.73)	1.17 (0.94–1.46)	1.32 (1.07–1.63)	
Yes	1 (reference)	0.86 (0.58–1.27)	0.8 (0.52–1.24)	0.64 (0.43–0.95)	0.96 (0.67–1.37)	
Smoking status						0.096
Never/former smoker	1 (reference)	0.90 (0.71–1.15)	1.18 (0.92–1.51)	0.96 (0.77–1.21)	1.13 (0.91–1.4)	
Current smoker	1 (reference)	1.33 (0.9–1.95)	1.3 (0.85–1.97)	1.12 (0.76–1.64)	1.47 (1.02–2.11)	
Education level						0.129
≤High school	1 (reference)	0.68 (0.46–1.02)	0.9 (0.61–1.35)	0.78 (0.55–1.1)	0.9 (0.65–1.25)	
≥College graduate	1 (reference)	1.3 (0.91–1.86)	1.61 (1.11–2.34)	1.21 (0.85–1.73)	1.37 (0.97–1.95)	
Obesity						0.985
BMI < 25	1 (reference)	1.03 (0.81–1.3)	1.26 (0.98–1.61)	1.06 (0.85–1.33)	1.25 (1.005–1.56)	
BMI ≥ 25	1 (reference)	1.003 (0.69–1.45)	1.1 (0.74–1.62)	0.95 (0.67–1.35)	1.09 (0.78–1.51)	
Model 2						
Sex						0.276
Male	1 (reference)	1.09 (0.86–1.38)	1.24 (0.97–1.59)	1.01 (0.8–1.26)	1.26 (1.02–1.57)	
Female	1 (reference)	0.84 (0.58–1.23)	1.11 (0.75–1.63)	1.04 (0.73–1.46)	1.18 (0.84–1.65)	
Regular exercise						0.038
No	1 (reference)	1.07 (0.85–1.35)	1.36 (1.07–1.73)	1.18 (0.95–1.46)	1.33 (1.08–1.65)	
Yes	1 (reference)	0.86 (0.58–1.27)	0.8 (0.52–1.25)	0.64 (0.43–0.96)	0.99 (0.69–1.42)	
Smoking status						0.092
Never/former smoker	1 (reference)	0.9 (0.71–1.15)	1.18 (0.92–1.51)	0.97 (0.77–1.21)	1.15 (0.92–1.42)	
Current smoker	1 (reference)	1.33 (0.9–1.95)	1.3 (0.85–1.98)	1.12 (0.77–1.64)	1.5 (1.05–2.16)	
Education level						0.129
≤High school	1 (reference)	0.69 (0.46–1.02)	0.91 (0.61–1.36)	0.78 (0.55–1.11)	0.92 (0.67–1.28)	
≥College graduate	1 (reference)	1.3 (0.91–1.87)	1.62 (1.11–2.35)	1.22 (0.86–1.73)	1.39 (0.98–1.97)	
Obesity						0.986
BMI < 25	1 (reference)	1.03 (0.81–1.3)	1.26 (0.98–1.61)	1.06 (0.85–1.33)	1.25 (1.005–1.56)	
BMI ≥ 25	1 (reference)	1.01 (0.7–1.46)	1.11 (0.75–1.64)	0.97 (0.68–1.37)	1.14 (0.82–1.58)	

Data are expressed as hazard ratios (95% confidence intervals). Abbreviations: BMI, body mass index. Model 1 was adjusted for sex, age, BMI (kg/m^2^, continuous), education level (university, community college graduate or higher), smoking status (smoker or nonsmoker), alcohol consumption (g/day), regular exercise. Model 2 was further adjusted for fasting glucose ≥ 126 mg/dL.

## Data Availability

The data that support the findings of this study are available from the corresponding author, Ki-Chul Sung, upon reasonable request.

## Supplementary Materials

The following supporting information can be downloaded at: https://www.mdpi.com/article/10.3390/jcm11195933/s1, Table S1: Baseline Characteristics of included and excluded participants; Table S2: Number of participants according to baseline HbA1c level; Table S3: Number of new-onset diabetes cases during the follow-up period according to HbA1c quintile; Table S4. All cancer-related mortality according to HbA1c level of participants (Age < 40); Table S5: All cancer-related mortality according to HbA1c level of participants (Age ≥ 40); Table S6: Specific cancer-related mortality according to HbA1c level of participants (Age < 40); Table S7: Specific cancer-related mortality according to HbA1c level of participants (Age ≥ 40).

## References

[1] Upadhyay J., Polyzos S.A., Perakakis N., Thakkar B., Paschou S.A., Katsiki N., Underwood P., Park K.H., Seufert J., Kang E.S. (2018). Pharmacotherapy of type 2 diabetes: An update. Metabolism.

[2] Sung H., Ferlay J., Siegel R.L., Laversanne M., Soerjomataram I., Jemal A., Bray F. (2021). Global Cancer Statistics 2020: GLOBOCAN Estimates of Incidence and Mortality Worldwide for 36 Cancers in 185 Countries. CA Cancer J. Clin..

[3] Jung K.-W., Won Y.-J., Hong S., Kong H.-J., Lee E.S. (2020). Prediction of Cancer Incidence and Mortality in Korea, 2020. Cancer Res. Treat..

[4] Edwards C.M., Cusi K. (2016). Prediabetes: A Worldwide Epidemic. Endocrinol. Metab. Clin. N. Am..

[5] Jung C.-H., Son J.W., Kang S., Kim W.J., Kim H.-S., Kim H.S., Seo M., Shin H.-J., Lee S.-S., Jeong S.J. (2021). Diabetes Fact Sheets in Korea, 2020: An Appraisal of Current Status. Diabetes Metab. J..

[6] Selvin E. (2021). Hemoglobin A_1c_—Using Epidemiology to Guide Medical Practice: Kelly West Award Lecture 2020. Diabetes Care.

[7] Consensus Committee (2007). Consensus Statement on the Worldwide Standardization of the Hemoglobin A1C Measurement: The American Diabetes Association, European Association for the Study of Diabetes, International Federation of Clinical Chemistry and Laboratory Medicine, and the International Diabetes Federation. Diabetes Care.

[8] Welsh K.J., Kirkman M.S., Sacks D.B. (2016). Role of Glycated Proteins in the Diagnosis and Management of Diabetes: Research Gaps and Future Directions. Diabetes Care.

[9] International Expert Committee (2009). International Expert Committee Report on the Role of the A1C Assay in the Diagnosis of Diabetes. Diabetes Care.

[10] Giovannucci E., Harlan D.M., Archer M.C., Bergenstal R.M., Gapstur S.M., Habel L.A., Pollak M., Regensteiner J.G., Yee D. (2010). Diabetes and cancer: A consensus report. CA Cancer J. Clin..

[11] De Beer J., Liebenberg L. (2014). Does cancer risk increase with HbA 1c, independent of diabetes?. Br. J. Cancer.

[12] Goto A., Noda M., Sawada N., Kato M., Hidaka A., Mizoue T., Shimazu T., Yamaji T., Iwasaki M., Sasazuki S. (2016). High hemoglobin A1c levels within the non-diabetic range are associated with the risk of all cancers. Int. J. Cancer.

[13] Rentsch C.T., Farmer R.E., Eastwood S.V., Mathur R., Garfield V., Farmaki A.-E., Bhaskaran K., Chaturvedi N., Smeeth L. (2020). Risk of 16 cancers across the full glycemic spectrum: A population-based cohort study using the UK Biobank. BMJ Open Diabetes Res. Care.

[14] Lu J., He J., Li M., Tang X., Hu R., Shi L., Su Q., Peng K., Xu M., Xu Y. (2019). Predictive Value of Fasting Glucose, Postload Glucose, and Hemoglobin A_1c_ on Risk of Diabetes and Complications in Chinese Adults. Diabetes Care.

[15] Siddiqui A.A., Spechler S.J., Huerta S., Dredar S., Little B.B., Cryer B. (2008). Elevated HbA1c Is an Independent Predictor of Aggressive Clinical Behavior in Patients with Colorectal Cancer: A Case-Control Study. Dig. Dis. Sci..

[16] De Bruijn K.M.J., Arends L.R., Hansen B.E., Leeflang S., Ruiter R., van Eijck C.H.J. (2013). Systematic review and meta-analysis of the association between diabetes mellitus and incidence and mortality in breast and colorectal cancer. Br. J. Surg..

[17] Huang Y., Zheng H., Chen P., Yang J., Lin S., Liu T., Chen S., Lu S., Chen J., Chen W. (2017). An Elevated HbA1c Level Is Associated With Short-Term Adverse Outcomes in Patients With Gastrointestinal Cancer and Type 2 Diabetes Mellitus. J. Clin. Med. Res..

[18] Bonagiri P.R., Shubrook J.H. (2020). Review of Associations Between Type 2 Diabetes and Cancer. Clin. Diabetes.

[19] Bancks M.P., Odegaard A.O., Pankow J.S., Koh W.P., Yuan J.M., Gross M.D., Pereira M.A. (2014). Glycated hemoglobin and all-cause and cause-specific mortality in Singaporean Chinese without diagnosed diabetes: The Singapore Chinese Health Study. Diabetes Care.

[20] Islam Z., Akter S., Inoue Y., Hu H., Kuwahara K., Nakagawa T., Honda T., Yamamoto S., Okazaki H., Miyamoto T. (2021). Prediabetes, Diabetes, and the Risk of All-Cause and Cause-Specific Mortality in a Japanese Working Population: Japan Epidemiology Collaboration on Occupational Health Study. Diabetes Care.

[21] Hope C., Robertshaw A., Cheung K., Idris I., English E. (2016). Relationship between HbA1c and cancer in people with or without diabetes: A systematic review. Diabet. Med..

[22] Choe Y.J., Choe S.A., Cho S.I. (2018). Trends in Infectious Disease Mortality, South Korea, 1983–2015. Emerg. Infect. Dis..

[23] KIM Y.J., SHIM J.-S., CHOI C.-B., BAE S.-C. (2012). Mortality and Incidence of Malignancy in Korean Patients with Rheumatoid Arthritis. J. Rheumatol..

[24] Kim N.H., Chang Y., Lee S.R., Ryu S., Kim H.J. (2020). Glycemic Status, Insulin Resistance, and Risk of Pancreatic Cancer Mortality in Individuals With and Without Diabetes. Off. J. Am. Coll. Gastroenterol. ACG.

[25] Yang Y.J. (2019). An Overview of Current Physical Activity Recommendations in Primary Care. Korean J. Fam. Med..

[26] Ryan H., Trosclair A., Gfroerer J. (2012). Adult current smoking: Differences in definitions and prevalence estimates—NHIS and NSDUH, 2008. J. Environ. Public Health.

[27] Baur D.M., Klotsche J., Hamnvik O.P., Sievers C., Pieper L., Wittchen H.U., Stalla G.K., Schmid R.M., Kales S.N., Mantzoros C.S. (2011). Type 2 diabetes mellitus and medications for type 2 diabetes mellitus are associated with risk for and mortality from cancer in a German primary care cohort. Metabolism.

[28] Zhang M., Li X., Zhang X., Yang Y., Feng Z., Liu X. (2014). Association of serum hemoglobin A1c, C-peptide and insulin-like growth factor-1 levels with the occurrence and development of lung cancer. Mol. Clin. Oncol..

[29] Saydah S.H., Platz E.A., Rifai N., Pollak M.N., Brancati F.L., Helzlsouer K.J. (2003). Association of markers of insulin and glucose control with subsequent colorectal cancer risk. Cancer Epidemiol. Prev. Biomark..

[30] Platz E.A., Hankinson S.E., Rifai N., Colditz G.A., Speizer F.E., Giovannucci E. (1999). Glycosylated hemoglobin and risk of colorectal cancer and adenoma (United States). Cancer Causes Control.

[31] Rinaldi S., Rohrmann S., Jenab M., Biessy C., Sieri S., Palli D., Tumino R., Mattiello A., Vineis P., Nieters A. (2008). Glycosylated hemoglobin and risk of colorectal cancer in men and women, the European prospective investigation into cancer and nutrition. Cancer Epidemiol. Prev. Biomark..

[32] Joshu C.E., Prizment A.E., Dluzniewski P.J., Menke A., Folsom A.R., Coresh J., Yeh H.C., Brancati F.L., Platz E.A., Selvin E. (2012). Glycated hemoglobin and cancer incidence and mortality in the Atherosclerosis in Communities (ARIC) Study, 1990–2006. Int. J. Cancer.

[33] Silbernagel G., Grammer T.B., Winkelmann B.R., Boehm B.O., März W. (2011). Glycated Hemoglobin Predicts All-Cause, Cardiovascular, and Cancer Mortality in People Without a History of Diabetes Undergoing Coronary Angiography. Diabetes Care.

[34] Ramdass V., Caskey E., Sklarz T., Ajmeri S., Patel V., Balogun A., Pomary V., Hall J., Qari O., Tripathi R. (2021). Association Between Obesity and Cancer Mortality: An Internal Medicine Outpatient Clinic Perspective. J. Clin. Med. Res..

[35] American Diabetes Association (2021). 2. Classification and Diagnosis of Diabetes: Standards of Medical Care in Diabetes—2021. Diabetes Care.

[36] Zhou X.H., Qiao Q., Zethelius B., Pyörälä K., Söderberg S., Pajak A., Stehouwer C.D.A., Heine R.J., Jousilahti P., Ruotolo G. (2010). Diabetes, prediabetes and cancer mortality. Diabetologia.

[37] Plácido J., Ferreira J.V., de Oliveira F., Sant’Anna P., Monteiro-Junior R.S., Laks J., Deslandes A.C. (2019). Association among 2-min step test, functional level and diagnosis of dementia. Dement. Neuropsychol..

[38] Park J.H., Hong J.Y., Park Y.S., Kang G., Han K., Park J.O. (2021). Association of prediabetes, diabetes, and diabetes duration with biliary tract cancer risk: A nationwide cohort study. Metabolism.

[39] Yoon Y.S., Keum N., Zhang X., Cho E., Giovannucci E.L. (2015). Hyperinsulinemia, insulin resistance and colorectal adenomas: A meta-analysis. Metabolism.

[40] Avgerinos K.I., Spyrou N., Mantzoros C.S., Dalamaga M. (2019). Obesity and cancer risk: Emerging biological mechanisms and perspectives. Metabolism.

[41] Hardikar P.S., Joshi S.M., Bhat D.S., Raut D.A., Katre P.A., Lubree H.G., Jere A., Pandit A.N., Fall C.H.D., Yajnik C.S. (2012). Spuriously High Prevalence of Prediabetes Diagnosed by HbA_1c_ in Young Indians Partly Explained by Hematological Factors and Iron Deficiency Anemia. Diabetes Care.

[42] Cui A., Ding D., Li Y. (2021). Regulation of Hepatic Metabolism and Cell Growth by the ATF/CREB Family of Transcription Factors. Diabetes.

[43] Dewidar B., Kahl S., Pafili K., Roden M. (2020). Metabolic liver disease in diabetes—From mechanisms to clinical trials. Metabolism.

[44] Mossenta M., Busato D., Dal Bo M., Toffoli G. (2020). Glucose Metabolism and Oxidative Stress in Hepatocellular Carcinoma: Role and Possible Implications in Novel Therapeutic Strategies. Cancers.

[45] Cheung K.S., Chan E.W., Chen L., Seto W.K., Wong I.C.K., Leung W.K. (2019). Diabetes Increases Risk of Gastric Cancer After *Helicobacter pylori* Eradication: A Territory-Wide Study With Propensity Score Analysis. Diabetes Care.

[46] Chen S., Tao M., Zhao L., Zhang X. (2017). The association between diabetes/hyperglycemia and the prognosis of cervical cancer patients: A systematic review and meta-analysis. Medicine.

[47] Jousheghany F., Phelps J., Crook T., Hakkak R. (2016). Relationship between level of HbA1C and breast cancer. BBA Clin..

[48] Marrone M.T., Selvin E., Barber J.R., Platz E.A., Joshu C.E. (2019). Hyperglycemia, Classified with Multiple Biomarkers Simultaneously in Men without Diabetes, and Risk of Fatal Prostate Cancer. Cancer Prev. Res..

[49] Zhang Y.-B., Pan X.-F., Chen J., Cao A., Zhang Y.-G., Xia L., Wang J., Li H., Liu G., Pan A. (2020). Combined lifestyle factors, incident cancer, and cancer mortality: A systematic review and meta-analysis of prospective cohort studies. Br. J. Cancer.

[50] Xu Z., Taylor J.A. (2014). Genome-wide age-related DNA methylation changes in blood and other tissues relate to histone modification, expression and cancer. Carcinogenesis.

