# Supplementary material for: Association of Glycosylated Hemoglobin Level and Cancer-Related Mortality in Patients without Diabetes
Source: J Clin Med. 2022 Oct 8;11(19):5933. doi: 10.3390/jcm11195933 (PMC9570990; doi:10.3390/jcm11195933)
Supplement: Supplementary file 1 [file jcm-11-05933-s001.zip › jcm-1928527-supplementary.pdf]

Supplemental Table S1. Baseline Characteristics of included and excluded participants

	Missing data	Overall	Analysis Excluded	Analysis Included	*p* value
Characteristics[1]		627,315	37,858	589,457	
Age (years)		39.99±10.83	51.5±12.18	39.25±10.31	<0.001
Male, n (%)		326459 (52.04)	20,933 (55.29)	305,526 (51.83)	<0.001
BMI (kg/m2)	168	23.34±3.34	24.75±3.61	23.25±3.3	<0.001
Fasting glucose (mg/dL)	6	95.09±15.93	126.88±43.04	93.05±9.05	<0.001
red blood cell count (10^6/$mm^{3)}$	16	4.74±0.46	4.74±0.47	4.75±0.46	0.004
Alcohol amount grams (g/day)	42,922	5 (1-14)	4 (0-17)	5 (1-14)	<0.001
Regular exercise		92,922 (14.81)	7,263 (19.18)	85,659 (14.53)	<0.001
Current smoker (%)		130,158 (20.75)	8,030 (21.21)	122,128 (20.72)	<0.001
≥College graduate (%)		359,660 (57.33)	15,418 (40.73)	344,242 (58.4)	<0.001

Data are expressed as mean±standard deviation or median (interquartile range). Abbreviations: BMI, body mass index; [1]Baseline characteristics were compared among groups using ANOVA test for continuous variables and Pearson's Chi-square test for categorical variables.

Supplmental Table S2. Number of participants according to baseline HbA1c level.

HbA1c, %	Frequency	Percent
5.1	37,069	8
5.2	55,769	12.04
5.3	73,674	15.9
5.4	84,327	18.2
5.5	84,107	18.15
5.6	72,787	15.71
5.7	55,546	11.99

Abbreviation: HbA1c, glycated hemoglobin A1c.

Supplemental table S3. Number of new-onset diabetes cases during the follow-up period according to HbA1c quintile

HbA1c quintile	number	Diabetes (fasting glucose ≥126mg/dL)		Diabetes (HbA1c ≥ 6.5%)	
		No	Yes	No	Yes
3-	79,234	78,575 (99.17)	659 (0.83)	78,752 (99.39)	482 (0.61)
5.2-	127,430	126,282 (99.1)	1,148 (0.9)	126,558 (99.32)	872 (0.68)
5.4-	82,849	81,881 (98.83)	968 (1.17)	82,092 (99.09)	757 (0.91)
5.5-	153,392	150,981 (98.43)	2,411 (1.57)	151,454 (98.74)	1,938 (1.26)
5.7-	146,552	136,993 (93.48)	9,559 (6.52)	138,348 (94.4)	8,204 (5.6)
Total	589,457	574,712 (97.5)	14,745 (2.5)	577,204 (97.92)	12,253 (2.08)

Abbreviation: HbA1c, glycated hemoglobin A1c.

Supplemental Table S4. All cancer-related mortality according to HbA1c level of participants (Age < 40)

HbA1c category	Person-years	Cancer mortality	Incidence Rate (per 10^4 PY)	Age- and sex-adjusted HR (95% CI)	Model 1	Model 2
Total period	2,585,040.2	275	1.06 (0.95 - 1.2)			
Q1	441,605.02	52	1.18 (0.9 - 1.55)	1 (reference)	1 (reference)	1 (reference)
Q2	637,467.41	55	0.86 (0.66 - 1.12)	0.77 (0.53 - 1.12)	0.78 (0.53 - 1.14)	0.78 (0.53 - 1.14)
Q3	387,231.75	48	1.24 (0.93 - 1.64)	1.14 (0.77 - 1.7)	1.18 (0.79 - 1.76)	1.18 (0.79 - 1.76)
Q4	653,959.63	73	1.12 (0.89 - 1.4)	1.05 (0.73 - 1.51)	0.95 (0.65 - 1.38)	0.95 (0.65 - 1.39)
Q5	464,776.43	47	1.01 (0.76 - 1.35)	0.95 (0.64 - 1.42)	0.96 (0.64 - 1.46)	0.98 (0.64 - 1.48)
P value for trend				0.510	0.719	0.680
Excluding death within 1-year cases	2,225,621.3	263	1.18 (1.05 - 1.33)			
Q1	385,126.1	50	1.3 (0.98 - 1.71)	1 (reference)	1 (reference)	1 (reference)
Q2	549,218.77	52	0.95 (0.72 - 1.24)	0.76 (0.51 - 1.12)	0.76 (0.51 - 1.13)	0.76 (0.52 - 1.13)
Q3	331,921.66	46	1.39 (1.04 - 1.85)	1.15 (0.77 - 1.71)	1.2 (0.8 - 1.81)	1.2 (0.8 - 1.81)
Q4	559,884.42	72	1.29 (1.02 - 1.62)	1.08 (0.75 - 1.56)	0.98 (0.66 - 1.43)	0.98 (0.67 - 1.43)
Q5	399,470.31	43	1.08 (0.8 - 1.45)	0.91 (0.6 - 1.38)	0.93 (0.61 - 1.43)	0.94 (0.61 - 1.45)

P value for trend				0.547	0.735	0.696

Data are expressed as hazard ratios (95% confidence intervals). Model 1 was adjusted for sex, age, BMI(kg/m2, continuous), education level (university, community college graduate or higher), smoking status (smoker or nonsmoker), alcohol consumption(g/day), regular exercise Model 2 was further adjusted for fasting glucose ≥ 126mg/dL

Supplemental Table S5. All cancer-related mortality according to HbA1c level of participants (Age ≥ 40)

HbA1c category	Person-years	Cancer mortality	Incidence Rate (per 10^4 PY)	Age- and sex-adjusted HR (95% CI)	Model 1	Model 2
Total period	1,628,095.5	1,437	8.83 (8.38 - 9.29)			
Q1	174,476.6	118	6.76 (5.65 - 8.1)	1 (reference)	1 (reference)	1 (reference)
Q2	280,517.69	207	7.38 (6.44 - 8.46)	1.11 (0.88 - 1.39)	1.1 (0.87 - 1.39)	1.1 (0.87 - 1.39)
Q3	193,923.33	162	8.35 (7.16 - 9.74)	1.18 (0.93 - 1.5)	1.2 (0.94 - 1.54)	1.2 (0.94 - 1.54)
Q4	413,685.51	328	7.93 (7.12 - 8.83)	1.05 (0.85 - 1.3)	1.03 (0.83 - 1.29)	1.04 (0.83 - 1.29)
Q5	565,492.33	622	11 (10.17 - 11.9)	1.21 (0.99 - 1.47)	1.24 (1 - 1.53)	1.26 (1.02 - 1.55)
P value for trend				0.114	0.070	0.046
Excluding death within 1-year cases	1,418,860.2	1,371	9.66 (9.16 - 10.19)			
Q1	155,979.45	114	7.31 (6.08 - 8.78)	1 (reference)	1 (reference)	1 (reference)
Q2	246,899.67	203	8.22 (7.17 - 9.43)	1.14 (0.9 - 1.43)	1.12 (0.88 - 1.42)	1.12 (0.88 - 1.42)
Q3	169,296.23	156	9.21 (7.88 - 10.78)	1.19 (0.94 - 1.52)	1.2 (0.93 - 1.54)	1.2 (0.94 - 1.54)
Q4	358,694	312	8.7 (7.78 - 9.72)	1.05 (0.85 - 1.31)	1.03 (0.82 - 1.29)	1.03 (0.82 - 1.29)
Q5	487,990.83	586	12.01 (11.07 - 13.02)	1.21 (0.99 - 1.49)	1.22 (0.98 - 1.51)	1.24 (1 - 1.54)
P value for trend				0.153	0.143	0.094

Data are expressed as hazard ratios (95% confidence intervals). Model 1 was adjusted for sex, age, BMI (kg/m2, continuous), education level (university, community college graduate or higher), smoking status (smoker or nonsmoker), alcohol consumption (g/day), regular exercise. Model 2 was further adjusted for fasting glucose ≥ 126mg/dL

Supplemental Table S6. Specific cancer-related mortality according to HbA1c level of participants (Age < 40)

HbA1c category	Person-years	Cancer mortality	Incidence Rate (per 10^4 PY)	Age- and sex adjusted HR (95% CI)	Model 1	Model 2
Lung cancer	2,585,040.2	28	0.11 (0.07 - 0.16)			
Q1	441,605.02	9	0.2 (0.11 - 0.39)	1 (reference)	1 (reference)	1 (reference)
Q2	637,467.41	5	0.08 (0.03 - 0.19)	0.41 (0.14 - 1.22)	0.41 (0.14 - 1.24)	0.41 (0.14 - 1.24)
Q3	387,231.75	6	0.15 (0.07 - 0.34)	0.83 (0.29 - 2.35)	0.85 (0.3 - 2.42)	0.86 (0.3 - 2.43)
Q4	653,959.63	3	0.05 (0.01 - 0.14)	0.25 (0.07 - 0.93)	0.17 (0.04 - 0.81)	0.17 (0.04 - 0.82)
Q5	464,776.43	5	0.11 (0.04 - 0.26)	0.59 (0.19 - 1.78)	0.62 (0.2 - 1.92)	0.65 (0.21 - 2)
P value for trend				0.217	0.197	0.216
Colorectal cancer	2,585,040.2	23	0.09 (0.06 - 0.13)			
Q1	441,605.02	3	0.07 (0.02 - 0.21)	1 (reference)	1 (reference)	1 (reference)
Q2	637,467.41	4	0.06 (0.02 - 0.17)	0.91 (0.2 - 4.08)	0.94 (0.21 - 4.22)	0.94 (0.21 - 4.2)
Q3	387,231.75	3	0.08 (0.02 - 0.24)	1.14 (0.23 - 5.65)	1.21 (0.24 - 6.01)	1.2 (0.24 - 5.95)
Q4	653,959.63	7	0.11 (0.05 - 0.22)	1.56 (0.4 - 6.07)	1.44 (0.36 - 5.8)	1.41 (0.35 - 5.71)
Q5	464,776.43	6	0.13 (0.06 - 0.29)	1.83 (0.45 - 7.43)	1.97 (0.48 - 8.06)	1.81 (0.44 - 7.54)
P value for trend				0.226	0.234	0.290
Liver cancer	2,585,040.2	9	0.03 (0.02 - 0.07)			
Q1	441,605.02	3	0.07 (0.02 - 0.21)	1 (reference)	1 (reference)	1 (reference)
Q2	637,467.41	2	0.03 (0.01 - 0.13)	0.51 (0.09 - 3.06)	0.53 (0.09 - 3.18)	0.53 (0.09 - 3.19)
Q3	387,231.75	3	0.08 (0.02 - 0.24)	1.29 (0.26 - 6.47)	0.93 (0.15 - 5.66)	0.93 (0.15 - 5.7)
Q4	653,959.63	1	0.02 (0 - 0.11)	0.26 (0.03 - 2.55)	0.29 (0.03 - 2.86)	0.29 (0.03 - 2.89)
Q5	464,776.43	-	N/A	N/A	N/A	N/A
P value for trend				0.128	0.139	0.150
Stomach cancer	2,585,040.2	47	0.18 (0.14 - 0.24)			
Q1	441,605.02	11	0.25 (0.14 - 0.45)	1 (reference)	1 (reference)	1 (reference)
Q2	637,467.41	7	0.11 (0.05 - 0.23)	0.44 (0.17 - 1.13)	0.47 (0.18 - 1.2)	0.47 (0.18 - 1.21)
Q3	387,231.75	6	0.15 (0.07 - 0.34)	0.62 (0.23 - 1.67)	0.58 (0.2 - 1.67)	0.58 (0.2 - 1.68)
Q4	653,959.63	15	0.23 (0.14 - 0.38)	0.9 (0.41 - 1.98)	0.93 (0.41 - 2.09)	0.93 (0.41 - 2.1)
Q5	464,776.43	8	0.17 (0.09 - 0.34)	0.66 (0.26 - 1.66)	0.74 (0.28 - 1.94)	0.76 (0.29 - 1.99)
P value for trend				0.998	0.909	0.870

HbA1c category	Person-years	Cancer mortality	Incidence Rate (per 10^4 PY)	Age- and sex adjusted HR (95% CI)	Model 1	Model 2
Prostate cancer†	2,585,040.2	0	N/A			
Q1	441,605.02	0	N/A	N/A	N/A	N/A
Q2	637,467.41	0	N/A	N/A	N/A	N/A
Q3	387,231.75	0	N/A	N/A	N/A	N/A
Q4	653,959.63	0	N/A	N/A	N/A	N/A
Q5	464,776.43	0	N/A	N/A	N/A	N/A
P value for trend						
Breast cancer‡	2,585,040.2	25	0.1 (0.07 - 0.14)	1 (reference)	1 (reference)	1 (reference)
Q1	441,605.02	3	0.07 (0.02 - 0.21)	1.18 (0.28 - 4.93)	1.18 (0.28 - 4.94)	1.18 (0.28 - 4.94)
Q2	637,467.41	5	0.08 (0.03 - 0.19)	0.84 (0.14 - 5.03)	0.85 (0.14 - 5.11)	0.85 (0.14 - 5.11)
Q3	387,231.75	2	0.05 (0.01 - 0.21)	2.59 (0.71 - 9.46)	1.61 (0.4 - 6.51)	1.62 (0.4 - 6.52)
Q4	653,959.63	10	0.15 (0.08 - 0.28)	1.89 (0.45 - 7.97)	2.07 (0.49 - 8.85)	2.1 (0.49 - 8.96)
Q5	464,776.43	5	0.11 (0.04 - 0.26)	0.128	0.258	0.251
P value for trend						
Cervical cancer‡	2,585,040.2	7	0.03 (0.01 - 0.06)			
Q1	441,605.02	2	0.05 (0.01 - 0.18)	1 (reference)	1 (reference)	1 (reference)
Q2	637,467.41	1	0.02 (0 - 0.11)	0.33 (0.03 - 3.64)	0.33 (0.03 - 3.59)	0.33 (0.03 - 3.59)
Q3	387,231.75	0	N/A	N/A	N/A	N/A
Q4	653,959.63	1	0.02 (0 - 0.11)	0.33 (0.03 - 3.64)	0.31 (0.03 - 3.49)	0.31 (0.03 - 3.5)
Q5	464,776.43	3	0.06 (0.02 - 0.2)	1.39 (0.23 - 8.6)	0.81 (0.11 - 6.05)	0.82 (0.11 - 6.12)
P value for trend				0.623	0.867	0.875
Pancreatic cancer	2,585,040.2	24	0.09 (0.06 - 0.14)			
Q1	441,605.02	5	0.11 (0.05 - 0.27)	1 (reference)	1 (reference)	1 (reference)
Q2	637,467.41	3	0.05 (0.02 - 0.15)	0.47 (0.11 - 1.99)	0.59 (0.13 - 2.63)	0.59 (0.13 - 2.65)
Q3	387,231.75	4	0.1 (0.04 - 0.28)	1.14 (0.3 - 4.27)	1.39 (0.34 - 5.57)	1.4 (0.35 - 5.63)
Q4	653,959.63	8	0.12 (0.06 - 0.24)	1.44 (0.46 - 4.45)	1.72 (0.51 - 5.81)	1.75 (0.52 - 5.92)
Q5	464,776.43	4	0.09 (0.03 - 0.23)	1.02 (0.27 - 3.87)	0.84 (0.18 - 3.88)	0.92 (0.2 - 4.22)
P for trend				0.398	0.500	0.434

Data are expressed as hazard ratios (95% confidence intervals). Model 1 was adjusted for sex, age, BMI (kg/m2, continuous), education level (university, community college graduate or higher), smoking status (smoker or nonsmoker), alcohol consumption (g/day), regular exercise. Model 2 was further adjusted for fasting glucose ≥ 126 mg/dL; †Male only; ‡Female only.

Supplemental Table S7. Specific cancer-related mortality according to HbA1c level of participants (Age ≥ 40)

HbA1c category	Person-years	Cancer mortality	Incidence Rate (per 10^4 PY)	Age- and sex-adjusted HR (95% CI)	Model 1	Model 2
Lung cancer	1,628,095.5	344	2.11 (1.9 - 2.35)			
Q1	174,476.6	18	1.03 (0.65 - 1.64)	1 (reference)	1 (reference)	1 (reference)
Q2	280,517.69	42	1.5 (1.11 - 2.03)	1.52 (0.87 - 2.64)	1.48 (0.85 - 2.57)	1.48 (0.85 - 2.57)
Q3	193,923.33	37	1.91 (1.38 - 2.63)	1.79 (1.02 - 3.14)	1.71 (0.97 - 3.03)	1.72 (0.97 - 3.03)
Q4	413,685.51	69	1.67 (1.32 - 2.11)	1.46 (0.87 - 2.46)	1.4 (0.83 - 2.37)	1.4 (0.83 - 2.38)
Q5	565,492.33	178	3.15 (2.72 - 3.65)	2.3 (1.41 - 3.76)	2.18 (1.33 - 3.59)	2.21 (1.34 - 3.64)
P value for trend				<0.001	0.001	0.001
Colorectal cancer	1,628,095.5	95	0.58 (0.48 - 0.71)			
Q1	174,476.6	2	0.11 (0.03 - 0.46)	1 (reference)	1 (reference)	1 (reference)
Q2	280,517.69	16	0.57 (0.35 - 0.93)	5.13 (1.18 - 22.32)	4.5 (1.02 - 19.82)	4.53 (1.03 - 19.95)
Q3	193,923.33	7	0.36 (0.17 - 0.76)	3.07 (0.64 - 14.78)	2.21 (0.43 - 11.41)	2.24 (0.43 - 11.56)
Q4	413,685.51	22	0.53 (0.35 - 0.81)	4.2 (0.98 - 17.92)	3.13 (0.72 - 13.7)	3.2 (0.73 - 14)
Q5	565,492.33	48	0.85 (0.64 - 1.13)	5.4 (1.3 - 22.45)	4.92 (1.17 - 20.64)	5.34 (1.27 - 22.41)
P value for trend				0.046	0.051	0.030
Liver cancer	1,628,095.5	206	1.27 (1.1 - 1.45)			
Q1	174,476.6	30	1.72 (1.2 - 2.46)	1 (reference)	1 (reference)	1 (reference)
Q2	280,517.69	43	1.53 (1.14 - 2.07)	0.91 (0.57 - 1.46)	0.85 (0.52 - 1.39)	0.85 (0.52 - 1.39)
Q3	193,923.33	27	1.39 (0.95 - 2.03)	0.78 (0.46 - 1.32)	0.74 (0.43 - 1.29)	0.75 (0.43 - 1.29)
Q4	413,685.51	47	1.14 (0.85 - 1.51)	0.61 (0.39 - 0.97)	0.64 (0.39 - 1.02)	0.64 (0.4 - 1.03)
Q5	565,492.33	59	1.04 (0.81 - 1.35)	0.5 (0.32 - 0.78)	0.47 (0.29 - 0.76)	0.48 (0.3 - 0.78)
P value for trend				<0.001	0.001	0.001
Stomach cancer	1,628,095.5	125	0.77 (0.64 - 0.91)			
Q1	174,476.6	7	0.4 (0.19 - 0.84)	1 (reference)	1 (reference)	1 (reference)

HbA1c category	Person-years	Cancer mortality	Incidence Rate (per 10^4 PY)	Age- and sex-adjusted HR (95% CI)	Model 1	Model 2
Q2	280,517.69	10	0.36 (0.19 - 0.66)	0.91 (0.35 - 2.39)	0.96 (0.34 - 2.71)	0.97 (0.34 - 2.72)
Q3	193,923.33	21	1.08 (0.71 - 1.66)	2.61 (1.11 - 6.16)	3.17 (1.28 - 7.88)	3.19 (1.28 - 7.91)
Q4	413,685.51	37	0.89 (0.65 - 1.23)	2.05 (0.91 - 4.63)	2.25 (0.94 - 5.42)	2.27 (0.94 - 5.46)
Q5	565,492.33	50	0.88 (0.67 - 1.17)	1.74 (0.78 - 3.89)	2.18 (0.92 - 5.17)	2.25 (0.94 - 5.35)
P value for trend				0.094	0.031	0.023
Prostate cancer†	1,628,095.5	25	0.15 (0.1 - 0.23)			
Q1	174,476.6	0	N/A	N/A	N/A	N/A
Q2	280,517.69	2	0.07 (0.02 - 0.29)	N/A	N/A	N/A
Q3	193,923.33	4	0.21 (0.08 - 0.55)	N/A	N/A	N/A
Q4	413,685.51	8	0.19 (0.1 - 0.39)	N/A	N/A	N/A
Q5	565,492.33	11	0.19 (0.11 - 0.35)	N/A	N/A	N/A
P value for trend						
Breast cancer‡	1,628,095.5	45	0.28 (0.21 - 0.37)			
Q1	174,476.6	4	0.23 (0.09 - 0.61)	1 (reference)	1 (reference)	1 (reference)
Q2	280,517.69	7	0.25 (0.12 - 0.52)	0.98 (0.29 - 3.35)	1.12 (0.28 - 4.49)	1.12 (0.28 - 4.49)
Q3	193,923.33	8	0.41 (0.21 - 0.82)	1.62 (0.49 - 5.39)	1.6 (0.4 - 6.42)	1.6 (0.4 - 6.43)
Q4	413,685.51	12	0.29 (0.16 - 0.51)	1.11 (0.36 - 3.47)	1.23 (0.34 - 4.52)	1.23 (0.34 - 4.53)
Q5	565,492.33	14	0.25 (0.15 - 0.42)	0.92 (0.3 - 2.87)	1.09 (0.3 - 3.96)	1.11 (0.3 - 4.05)
P value for trend				0.780	0.981	0.981
Cervical cancer‡	1,628,095.5	9	0.06 (0.03 - 0.11)			
Q1	174,476.6	1	0.06 (0.01 - 0.41)	1 (reference)	1 (reference)	1 (reference)
Q2	280,517.69	2	0.07 (0.02 - 0.29)	1.06 (0.1 - 11.74)	N/A	N/A
Q3	193,923.33	1	0.05 (0.01 - 0.37)	0.74 (0.05 - 11.87)	N/A	N/A
Q4	413,685.51	1	0.02 (0.003 - 0.17)	0.32 (0.02 - 5.26)	N/A	N/A
Q5	565,492.33	4	0.07 (0.03 - 0.19)	0.86 (0.09 - 8.2)	N/A	N/A
P value for trend				0.727	0.837	0.885
Pancreatic cancer	1,628,095.5	142	0.87 (0.74 - 1.03)			
Q1	174,476.6	7	0.4 (0.19 - 0.84)	1 (reference)	1 (reference)	1 (reference)
Q2	280,517.69	20	0.71 (0.46 - 1.11)	1.85 (0.78 - 4.37)	1.75 (0.74 - 4.17)	1.75 (0.73 - 4.16)
Q3	193,923.33	17	0.88 (0.54 - 1.41)	2.18 (0.9 - 5.26)	2.06 (0.84 - 5.01)	2.04 (0.84 - 4.97)
Q4	413,685.51	29	0.7 (0.49 - 1.01)	1.66 (0.72 - 3.81)	1.47 (0.63 - 3.41)	1.45 (0.62 - 3.37)
Q5	565,492.33	69	1.22 (0.96 - 1.54)	2.44 (1.11 - 5.38)	2.36 (1.07 - 5.24)	2.25 (1.01 - 5)

HbA1c category	Person-years	Cancer mortality	Incidence Rate (per 10^4 PY)	Age- and sex-adjusted HR (95% CI)	Model 1	Model 2
P for trend				0.042	0.050	0.084

Data are expressed as hazard ratios (95% confidence intervals). Model 1 was adjusted for sex, age, BMI (kg/m2, continuous), education level (university, community college graduate or higher), smoking status (smoker or nonsmoker), alcohol consumption (g/day), regular exercise; Model 2 was further adjusted for fasting glucose ≥ 126 mg/dL; †Male only; ‡Female only.
